# Facioscapulohumeral muscular dystrophy and Charcot-Marie-Tooth neuropathy 1A - evidence for “double trouble” overlapping syndromes

**DOI:** 10.1186/1471-2350-14-92

**Published:** 2013-09-16

**Authors:** Olivia Schreiber, Peter Schneiderat, Wolfram Kress, Bernd Rautenstrauss, Jan Senderek, Benedikt Schoser, Maggie C Walter

**Affiliations:** 1 Friedrich-Baur-Institute, Department of Neurology, Ludwig-Maximilians-University Munich, Ziemssenstrasse 1, D-80336 Munich, Germany; 2 Department of Human Genetics, Julius-Maximilians-University Würzburg, Biozentrum, Am Hubland, D-97074 Würzburg, Germany; 3 Medizinisch Genetisches Zentrum, Bayerstrasse 3–5 D-80335 Munich, Germany

**Keywords:** Facioscapulohumeral muscular dystrophy, Charcot-Marie-Tooth neuropathy 1A, Hereditary motor and sensory neuropathy, Overlapping syndrome, Double trouble

## Abstract

**Background:**

We report on a patient with genetically confirmed overlapping diagnoses of CMT1A and FSHD. This case adds to the increasing number of unique patients presenting with atypical phenotypes, particularly in FSHD. Even if a mutation in one disease gene has been found, further genetic testing might be warranted in cases with unusual clinical presentation.

**Case presentation:**

The reported 53 years old male patient suffered from walking difficulties and foot deformities first noticed at age 20. Later on, he developed scapuloperoneal and truncal muscle weakness, along with atrophy of the intrinsic hand and foot muscles, pes cavus, claw toes and a distal symmetric hypoesthesia. Motor nerve conduction velocities were reduced to 20 m/s in the upper extremities, and not educible in the lower extremities, sensory nerve conduction velocities were not attainable. Electromyography showed both, myopathic and neurogenic changes. A muscle biopsy taken from the tibialis anterior muscle showed a mild myopathy with some neurogenic findings and hypertrophic type 1 fibers. Whole-body muscle MRI revealed severe changes in the lower leg muscles, tibialis anterior and gastrocnemius muscles were highly replaced by fatty tissue. Additionally, fatty degeneration of shoulder girdle and straight back muscles, and atrophy of dorsal upper leg muscles were seen. Taken together, the presenting features suggested both, a neuropathy and a myopathy. Patient’s family history suggested an autosomal dominant inheritance.

Molecular testing revealed both, a hereditary motor and sensory neuropathy type 1A (HMSN1A, also called Charcot-Marie-Tooth neuropathy 1A, CMT1A) due to a *PMP22* gene duplication and facioscapulohumeral muscular dystrophy (FSHD) due to a partial deletion of the D4Z4 locus (19 kb).

**Conclusion:**

Molecular testing in hereditary neuromuscular disorders has led to the identification of an increasing number of atypical phenotypes. Nevertheless, finding the right diagnosis is crucial for the patient in order to obtain adequate medical care and appropriate genetic counseling, especially in the background of arising curative therapies.

## Background

Hereditary motor and sensory neuropathy (HMSN), also called Charcot-Marie-Tooth (CMT) disease, is the most common inherited neuromuscular disorder with an estimated prevalence of 1:2,500 [1]. Roughly one third of all cases are caused by an autosomal dominant inherited 1.5 Megabase (Mb) tandem duplication encompassing the peripheral myelin protein 22 gene (*PMP22*) on chromosome 17p11.2-12 which encodes an important component of peripheral nervous system myelin [2-4]. Phenotypically, patients show a symmetric and distally pronounced muscle weakness and sensory deficits affecting the feet and lower legs and, to a lesser extent, the hands and forearms. Progressive muscle atrophy results in “steppage” gait and secondary foot deformities and can lead to severe disablement. Sensory deficits and paraesthesia are usually less prominent than in other neuropathies. Electrophysiology is an important tool for diagnosis and classification into demyelinating CMT1 (motor conduction velocities (MCVs) of median nerve <38 m/s) and axonal CMT2 (median nerve MCV > 38 m/s). Supportive treatment includes rehabilitative therapy and surgical treatment of skeletal deformities and soft-tissue abnormalities in a multidisciplinary approach [5,6]. Curative therapeutic options are under investigation.

Autosomal dominant facioscapulohumeral muscular dystrophy (FSHD) is the third most common muscular dystrophy with an estimated prevalence of about 1:20,000 in Europe [7]. The four main diagnostic criteria defining FSHD are (1) onset in facial or shoulder muscles with sparing of extraocular, pharyngeal and lingual muscles and the myocardium, (2) facial weakness in more than half of the affected family members, (3) autosomal dominant inheritance and (4) evidence of a myopathic disease in electromyography and muscle biopsy [8]. The causative gene defect in the large majority (>95%) of FSHD patients is a contraction of a repetitive element on chromosome 4q35 known as D4Z4 to 1 to 10 units (in healthy individuals 11 to 100 units). Because FSHD is apparently not due to a conventional mutation within a neighboring protein-coding gene, the exact pathophysiological mechanism of the repeat loss causing muscle disease is unknown [9]. The phenotypic spectrum is wide, even in the same family, showing a great variability in the level of impairment and disease progression. Therefore, it is still a diagnostic challenge to correctly diagnose FSHD since a broad clinical diversity results from a shortened D4Z4 locus.

With the advent of new causative therapeutic options finding a patient’s correct diagnosis is essential. The case presented here shows a new phenotype of two overlapping neuromuscular diseases which emphasizes the diagnostic challenges.

## Case presentation

### Methods

The patient was clinically and electrophysiologically examined by the authors.

An open muscle biopsy was performed at the age of 21 after first clinical symptoms occurred. Standard histological examination was carried out as described previously [10] and the muscle biopsy specimens were revisited after disease progression.

Whole-body muscle magnetic resonance imaging (MRI) was performed using a 3.0-T MR system. A routine muscular protocol containing axial T1-weighted (T1w) spin echo sequences was used (slice thickness: 6 mm). The protocol additionally included coronal and axial planes at four levels covering the whole body. The left distal pelvic muscles and proximal femoral muscles could not be examined as the patient had a hip implant.

Using MLPA (Multiplex Ligation-dependent Probe Amplification)-analysis (MRC-Holland P033) the *PMP22* gene was searched for deletions and duplications. FSHD diagnosis was established by Southern blot analysis for the D4Z4 locus as reported elsewhere [11]. Furthermore, the mutation-hot spot region of the myotilin gene was examined by direct sequencing of exon 2 (NM_006790).

This case report was exempt as part of the patient’s standard care. The patient consented to all performed diagnostic analyses as part of a standard diagnostic work-up and care as well as the publication of his clinical data, images (clinical photographs, MRI, muscle biopsy) and videos. However, he preferred to have his face made unrecognizable on the photos. Additionally, his family members gave permission for publication of their medical histories.

#### Clinical data

Childhood and adolescence were reported normal. First clinical symptoms were noted at age 20 with pes cavus, claw toes and walking difficulties. During the 5th decade, weakness of arm elevation and distal weakness of hands affecting the dorsal interossei with reduced abduction in the metacarpophalangeal (MCP) joints and a weakness in thumb abduction occurred. The patient reported mild hypoesthesia of fingers and toes and he had marked difficulties to button up his shirt. He walked unaided and maximum walking distance was not impaired.

**Figure 1:**
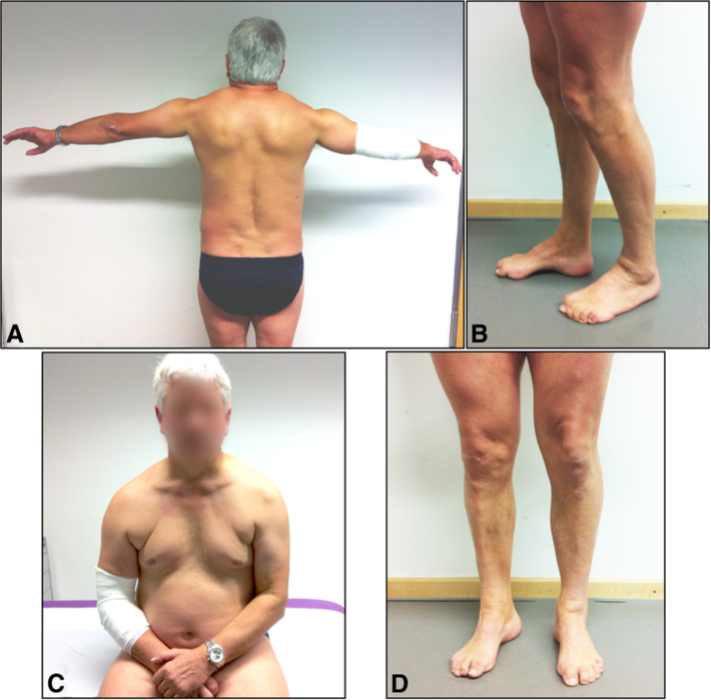
**Clinical phenotype.** The patient shows a scapuloperoneal pattern of muscle wasting and weakness with pectoral wasting and scapular winging **(A, C)**. The arms cannot be lifted over the horizontal level **(A)**. At the lower extremities, lower leg atrophy **(D)**, pes cavus and claw toes are pronounced **(B)**.

Clinical examination revealed a scapuloperoneal phenotype associated with pectoralis muscle atrophy, truncal weakness and scapular winging. The patient was not able to lift his arms above the horizontal level. There was no facial and bulbar weakness and no Beevor’s sign. He did not suffer from scoliosis or contractures. Standing or walking on toes or heels was not possible and in heel to toe walking (tandem gait) an unsteady gait, probably due to sensory loss, was observed. Additionally, lower limb weakness (extensor digitorum MRC 4/5, abductor pollicis MRC 4/5, tibialis anterior and gastrocnemius muscles MRC 2/5) along with marked muscle atrophy was found. Distal symmetric hypoesthesia and the described foot deformities were observed (Figure 1). Deep tendon reflexes were absent. Needle EMG showed mild myopathic changes in proximal muscles such as M. biceps brachii and M. iliopsoas and neurogenic changes in tibialis anterior muscles. Nerve conduction velocities in upper limb motor nerves showed a demyelinating neuropathy (median nerve 17 m/s, DML 9.5 ms; ulnar nerve 21 m/s, DML 7.2 ms). Compound motor action potentials (CMAPs) were normal in amplitude without signs of conduction block or temporal dispersion. Lower limb motor nerves were not educible. Sensory nerve conduction velocities were repeatedly not attainable. CK levels were elevated up to 300 U/l (normal value < 180).

In summary, the patient presented with a scapuloperoneal weakness and atrophy, foot deformities along with sensorimotor demyelinating neuropathy and mild hyerCKemia.

#### Family history

**Figure 2:**
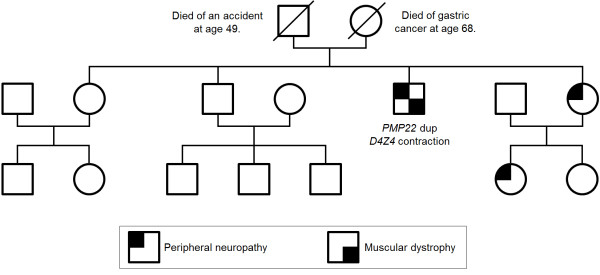
**Family history.** The patient’s parents died early and had no symptoms of muscle weakness or neuropathy. Neither did the mother’s four siblings and their progenies. Besides our patient one of his sisters and her older daughter show similar symptoms of peripheral neuropathy with foot deformities and gait difficulties. No symptoms of muscle wasting or weakness occurred in these two family members until now.

The patient has three siblings (two sisters age 49 and 61, and one brother age 51). One sister and her daughter suffer from a sensorimotor neuropathy, but were not available for further investigations. The parents were reported healthy, however, the father died early at age 49 due to an accident and the mother died at age 68 due to gastric cancer. The mother’s four siblings and their progenies as well as the patient’s grandparents were reported healthy (Figure 2).

#### MRI

**Figure 3:**
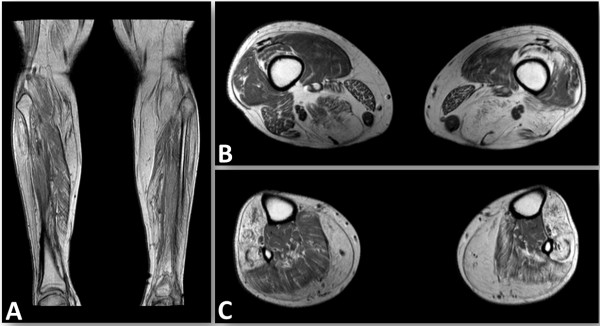
**MR imaging.** Whole-body MRI shows nearly complete fatty atrophy of the tibialis anterior muscles and the Mm. gastrocnemii, as well as moderate fatty replacement of muscle tissue in the soleus muscle **(A, C)**. Atrophy of the dorsal upper leg muscles is pronounced in the M. semimembranosus and M. biceps femoris **(B)**. Gluteal muscles revealed mild fatty degeneration. Additionally, fatty muscle degeneration of scapular fixators (Mm. serrati and Mm. latissimi dorsi) and axial back muscles was detected (data not shown). The pelvis and proximal upper legs were not examined as the patient had a hip implant.

The muscle MRI showed the most severe changes in the lower leg muscles. Tibialis anterior and gastrocnemius muscles were largely replaced by fatty tissue. Besides, fatty degeneration of serratus, latissimus dorsi and straight back muscles (data not shown) as well as atrophy of dorsal upper leg muscles, mainly biceps femoris and semimembranosus, were seen (Figure 3).

#### Muscle biopsy

**Figure 4:**
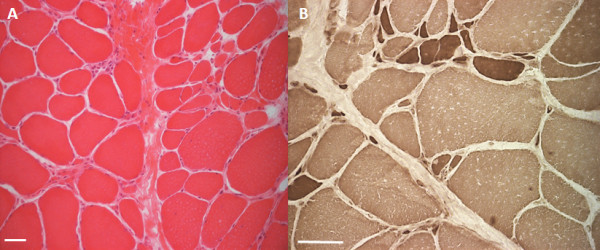
**Muscle biopsy. (A)** H&E staining of a muscle biopsy of the left anterior tibial muscle with mild myopathic changes indicated by muscle fiber splitting and increase in endomysial connective tissue. Additionally, in the ATPase pH 4.1 staining **(B)**, a type I fiber predominance without evidence of fiber type grouping and numerous hypertrophic type I fibers are notable. Bars in **A** and **B** adjusted to 50 μm.

An open muscle biopsy of the left tibialis anterior muscle was performed at the age of 21. It showed mild myopathic alterations with fiber splitting and increase of endomysial connective tissue, however some small neurogenic-like muscles fibers and numerous hypertrophic type I fibers were seen. This biopsy did not reveal any necrotic fibers or inflammatory changes (Figure 4A). ATPase staining revealed no fiber type grouping, but a type-1 fiber predominance of up to 80%, as commonly seen in anterior tibial muscle (Figure 4B). Trichrome staining showed no ragged red fibers. No myofibrillar or mitochondrial changes were detected.

#### Molecular analysis

Molecular analysis of the *PMP22* gene showed the typical duplication encompassing the *PMP22* gene on chromosome 17p11.2-12 confirming the diagnosis of CMT1A. No mutations were found in exon 2 of the myotilin gene. A shortened chromosome 4q-specific fragment of 19 kb in the D4Z4 locus (FSHD repeat) and the FSHD-related 4qA haplotype were observed leading to the diagnosis of FSHD.

## Discussion

Both hereditary sensorimotor neuropathy due to *PMP22* duplication (CMT1A) and facioscapulohumeral muscular dystrophy (FSHD) due to a shortened fragment of the D4Z4 locus (19 kb) were identified in the described patient. To the best of our knowledge this is one of only three published cases describing coincidence of CMT1A and FSHD.

Recent epidemiological data suggest that the frequency of CMT1A is around 1:7,500 [4] while the frequency of FSHD is estimated at 1:20,000 [7]. Thus, the chance of being affected by both disorders is about 1:150,000,000.

In the patient’s family history his sister and her daughter were suggestive of CMT1A as well. However, since both parents have died and no DNA samples of further family members were available, segregation could not be proven. Our patient showed typical symptoms of this generalized, primarily demyelinating neuropathy with reduced nerve conduction velocities, distal hypoesthesia and distal muscle atrophy leading to steppage gait and foot deformities like pes cavus und claw toes [1].

Since additional features like proximal muscle weakness of the shoulder girdle, myopathic changes in muscle biopsy specimen and EMG analysis were suggestive of additional primary involvement of the skeletal muscle genetic analyses were extended to include frequent forms of muscular dystrophies and myopathies. FSHD diagnosis was established by Southern blot analysis for the D4Z4 locus showing contraction of the repeat (19 kb) and a FSHD-related 4qA haplotype. It remains unclear if FSHD results from a *de novo* mutation occurring in 10% to 30% of all cases [12] or from familial inheritance as we cannot rule out the possibility of an undiagnosed mild presentation in other family members. Until now, the pathogenic relevance of the D4Z4 deletion has not been fully clarified and has been questioned in recent studies [13]. However, in addition to the clinical findings, electromyography in our patient revealed mild myopathic changes in proximal muscles along with the myopathic changes in the muscle biopsy specimen. Along with shortening of the D4Z4 locus to 19 kb on *EcoRI* + *BlnI* double digestion, FSHD is likely to be causative for the additional myopathy.

The classical FSHD phenotype was described in 1884 by Landouzy and Dejerine with facial weakness, shoulder girdle and pectoral muscle weakness and atrophy, in some cases resulting in subsequent impairment of pelvic and lower leg muscles [14]. But clinical variability has long been recognized in neuromuscular disorders. This has become most obvious with the advent of molecular genetic testing showing that identical molecular defects can result in diverse clinical presentations. This is especially true for FSHD where several unusual phenotypes and atypical morphological features ranging from hyperCKemia, severe muscle pain, facial-sparing, hypertrophic cardiomyopathy, camptocormia, distal and axial myopathy, along with atypical morphological changes such as vacuolar and/or inflammatory myopathy, nemaline rods, and deficiency of complex III of the mitochondrial respiratory chain were reported [15-23]. Our patient provides further evidence for this variability by not fulfilling all four major diagnostic criteria for diagnosis of FSHD as described above [8]. Autosomal dominant inheritance cannot be proven since the parents were not available for molecular testing, and facial weakness was not seen.

Interfamilial and intrafamilial clinical variability has also been observed in CMT [24,25] including cases of demyelinating CMT with scapuloperoneal distribution of motor impairment reported in the 1980s [26,27]. In contrast to our case, these cases were confined to clinical findings since molecular genetic testing was not yet available in the 1980s. Harding and Thomas described a constellation of clinical symptoms similar to our patient but with striking wasting of both deltoid muscles which is rather uncommon in FSHD so that we cannot exclude that this patient suffered from another scapuloperoneal myopathy.

In addition to stochastic effects, environmental influences, allelic variation, modifier genes, somatic mosaicism and complex genetic and environmental interactions [28], some of this variability might be caused by concomitant mutations in other genes for neuromuscular conditions. In FSHD, such overlapping syndromes have been described several times in literature. Association with pathogenic mutations in other genes have been reported in cases of patients with mitochondrial myopathy/FSHD, Becker muscular dystrophy/FSHD, Duchenne muscular dystrophy/FSHD, Leber’s hereditary optic neuropathy/FSHD and caveolinopathy/FSHD determining overlapping phenotypes [15,29-33]. For CMT1A, concomitant mutations in the *PMP22* gene and the Connexin32 gene (causing CMTX), the DMPK1 gene (DM1 myotonic dystrophy) and the ABCD1 gene (adrenomyeloneuropathy) have been described to produce peculiar phenotypes [34]. In addition, a combination of CMTX with Becker muscular dystrophy has been reported [35], causing both, generalized weakness and CK elevation as typical phenotype for a muscular dystrophy as well as foot deformity, decreased tendon reflexes and sensory loss due to a mutation in the Connexin32 gene.

There is one further report similar to ours on one family suffering from CMT1A and FSHD [36]. Auer-Grumbach et al. reported on this family affected by genetically confirmed CMT1A with scapuloperoneal motor deficit but conflicting data concerning the concomitant presence of FSHD. Co-segregation of FSHD in this family was unconvincing and haplotype analysis on chromosome 4q was not performed as it was not described in the paper. In contrast to our report, no muscle histology was performed and EMG revealed a mild chronic neurogenic pattern in proximal and distal muscles. However, our patient showed myopathic changes assessed by electromyography and muscle biopsy which further support the molecular diagnosis of FSHD.

In our patient a contracted fragment of 19 kb in the D4Z4 locus was detected. It is generally accepted that there is a correlation between clinical severity and size of the D4Z4 repeat: small repeats stand for early age at onset and severe clinical course, whereas larger repeats mean later age at onset and milder course [37]. Although the patient reported here carries a repeat size usually expected to result in a moderately severe phenotype with onset in the 1^st^ or 2^nd^ decade, he showed only mild symptoms of shoulder girdle weakness developing in the second half of life without facial or proximal leg muscle impairment until now. There is no obvious explanation for the unexpectedly mild phenotype in our patient and in contrast, the association of FSHD with X-linked CMT and Duchenne muscular dystrophy leads to severe infantile phenotypes [31,38]. Butefisch et al. described a further patient with the combination of CMT1A and FSHD in 1998 [39]. This female patient inherited CMT1A from her father and FSHD from her mother and this comorbidity resulted in severe generalized weakness, respiratory insufficiency and early death. However, according to the information given in the paper, only the diagnosis of CMT1A was proven by molecular testing but not the FSHD, neither in the patient nor in relatives. Thus, we cannot exclude the possibility that another progressive muscular dystrophy might be causative for this devastating clinical course in this patient.

All in all, too few “double trouble” patients are known to draw significant conclusions on how concomitant genetic conditions modify each other’s severity and progression in these patients. Nevertheless, the number of cases with genetically proven double trouble is likely to increase with the availability of improved genetic testing and whole exome or genome sequencing methods. This will result in an even broader variety of atypical clinical pictures but may also help to understand interference at the phenotypic, genetic and epigenetic levels having a direct impact on medical care and genetic counseling.

## Conclusion

Our case reports on a patient with genetically confirmed overlapping diagnoses of CMT1A and FSHD. It adds to the increasing number of unique patients presenting with atypical and overlapping phenotypes, particularly in FSHD. Even if a mutation in a disease gene has been found, further genetic testing might be warranted in cases with unusual clinical presentation having a direct impact on medical care and genetic counseling.

### Consent

This study was exempt as part of the patient’s standard care. The patient consented to all performed diagnostic analyses as part of a standard diagnostic work-up and care as well as the publication of his clinical data, images (clinical photographs, MRI, muscle biopsy) and videos. However, he preferred to have his face made unrecognizable on the photos. Additionally, his family members gave permission for publication of their medical histories. A copy of the written consent is available for review by the Editor of this journal.

## Competing interests

The authors declare that they have no competing interests.

## Authors’ contributions

OS, PS and MCW performed the clinical diagnostics of the patient. WK carried out the molecular genetic studies of the myotilin gene and the D4Z4 locus for FSHD. BR carried out the molecular genetic studies of the *PMP22* gene. JS has revised the manuscript critically for important intellectual content. BS carried out the further processing of the muscle biopsy and interpreted the results. OS and PS contributed equally to this work. All authors read and approved the final manuscript.

## Pre-publication history

The pre-publication history for this paper can be accessed here:

http://www.biomedcentral.com/1471-2350/14/92/prepub
